# Simulation as a practical and versatile tool for sample size calculation

**DOI:** 10.1186/1745-6215-14-S1-O99

**Published:** 2013-11-29

**Authors:** Richard Hooper

**Affiliations:** 1 Queen Mary University of London, London, UK

## Background

The ethical significance of sample size is enshrined in principles of Good Clinical Practice and in quality standards for reporting trial results, yet sample size is often one of the most ill-considered aspects of a grant application. Researchers are also battling with an increasing complexity in study design. With some adaptive trial designs, for example, analytical solutions to sample size calculation problems may simply be unattainable. Monte Carlo simulation offers a general alternative.

## Methods

A versatile tool using simulation to calculate sample size for given statistical power was developed as an add-on to a general statistics software package. This tool allows determination of sample size for any analysis under any statistical model that can be programmed in the host package, and uses a novel algorithm that is more efficient than previously published alternatives.

## Results

The software can cope with non-parametric tests, multi-level models, multiple testing, group sequential methods, adaptive trial designs, and more. The main limitation of simulation is its speed, but with efficient algorithms and ever-faster processors this does not hinder its use as a practical tool for sample size calculation.

## Conclusions

Simulation has a rough-and-ready reputation, and some statisticians may favour the elegance and wholesomeness of an analytical solution even when only approximate, but this is to prefer an exact, wrong answer to one that is accurate to any desired precision. Software tools using simulation offer a simple, accessible and versatile approach to sample size calculation that is easily validated by others such as statistical reviewers.

